# Diagnostic Value of Initial Chest CT Findings for the Need of ICU Treatment/Intubation in Patients with COVID-19

**DOI:** 10.3390/diagnostics10110929

**Published:** 2020-11-10

**Authors:** Laura Büttner, Annette Aigner, Florian Nima Fleckenstein, Christina Maria Hamper, Martin Jonczyk, Bernd Hamm, Oriane Scholz, Georg Böning

**Affiliations:** 1 Charité—Universitätsmedizin Berlin, Corporate Member of Freie Universität Berlin, Humboldt-Universität zu Berlin, and Berlin Institute of Health, Institute of Radiology, Charitéplatz 1, 10117 Berlin, Germany; florian.fleckenstein@charite.de (F.N.F.); christina-maria.hamper@charite.de (C.M.H.); martin.jonczyk@charite.de (M.J.); bernd.hamm@charite.de (B.H.); oriane.scholz@charite.de (O.S.); georg.boening@charite.de (G.B.); 2 Charité—Universitätsmedizin Berlin, Corporate Member of Freie Universität Berlin, Humboldt-Universität zu Berlin, and Berlin Institute of Health, Institute of Biometry and Clinical Epidemiology, Charitéplatz 1, 10117 Berlin, Germany; annette.aigner@charite.de; 3 Berlin Institute of Health (BIH), Anna-Louisa-Karsch 2, 10178 Berlin, Germany

**Keywords:** COVID-19, SARS-CoV-2, ventilation, CT, ICU, intubation

## Abstract

Computed tomography (CT) plays an important role in the diagnosis of COVID-19. The aim of this study was to evaluate a simple, semi-quantitative method that can be used for identifying patients in need of subsequent intensive care unit (ICU) treatment and intubation. We retrospectively analyzed the initial CT scans of 28 patients who tested positive for SARS-CoV-2 at our Level-I center. The extent of lung involvement on CT was classified both subjectively and with a simple semi-quantitative method measuring the affected area at three lung levels. Competing risks Cox regression was used to identify factors associated with the time to ICU admission and intubation. Their potential diagnostic ability was assessed with receiver operating characteristic (ROC)/area under the ROC curves (AUC) analysis. A 10% increase in the affected lung parenchyma area increased the instantaneous risk of intubation (hazard ratio (HR) = 2.00) and the instantaneous risk of ICU admission (HR 1.73). The semi-quantitative measurement outperformed the subjective assessment diagnostic ability (AUC = 85.6% for ICU treatment, 71.9% for intubation). This simple measurement of the involved lung area in initial CT scans of COVID-19 patients may allow early identification of patients in need of ICU treatment/intubation and thus help make optimal use of limited ICU/ventilation resources in hospitals.

## 1. Introduction

Severe acute respiratory syndrome coronavirus 2 (SARS-CoV-2) was first identified in December 2019 in Wuhan, China [1]. The main transmission route of the virus is via droplet infection [2]. The course of coronavirus disease 2019 (COVID-19) is unspecific and varies greatly, ranging from asymptomatic cases to severe pneumonia with lung failure and death [3]. In about 3–6% of COVID-19 patients, a critical or even life-threatening course has been reported [4]. Up to 17% of patients require ventilation [5]. Risk factors for a severe course include older age, smoking, and pre-existing conditions such as chronic lung diseases, or diabetes mellitus [4]. However, intensive care unit (ICU) capacity is limited and may even be exhausted in some countries. [6]. Changes in lung parenchyma known to occur in COVID-19 patients include ground-glass opacities, consolidations, and crazy paving. These changes can be diagnosed by computed tomography (CT) and characteristically show a peripheral distribution in the middle and lower lung regions [1,7,8]. A detailed description of CT findings in COVID-19 patients has been published by Chen et al. [7]. Approaches using artificial intelligence (AI) and volumetry confirm that quantification of pulmonary involvement may predict clinical outcome in terms of the need of ICU care [5,8]. However, tools like machine learning or volumetry are not available in all hospitals, and methods differ, making them difficult to compare results. Moreover, with rising prevalence rates in low- and middle-income countries, a simple method is needed [9]. The aim of this study was to evaluate a simple, semi-quantitative method for measuring pulmonary involvement in CT scans which can be used everywhere and may allow early identification of COVID-19 patients likely to require ICU treatment and intubation. Against the background of limited ICU and ventilation resources, our approach may be useful for making optimal use of available capacities.

## 2. Materials and Methods

**Figure 1:**
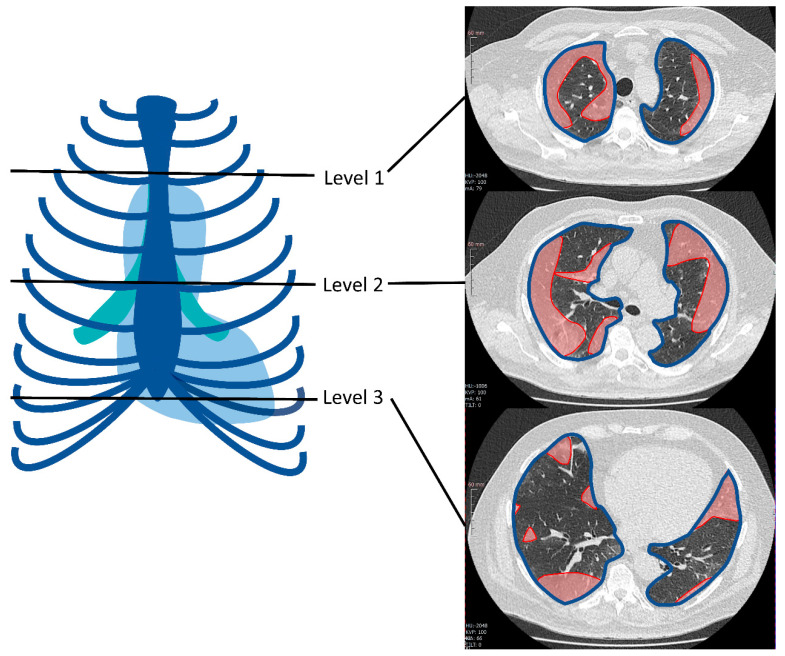
Example of regions of interest (ROI)-based semi-quantitative assessment of lung area involved in COVID-19 findings at the three levels chosen as standard: at the level of the aortic arch, at the level of the tracheal bifurcation, and at the inferior end of the xiphoid. Highlighted in red: area of involved lung parenchyma; the blue line outlines the total lung area in this section.

This retrospective study was approved by the Local Ethics Committee of our hospital (EA4/074/20). Informed consent was waived because of the retrospective study design. We analyzed the results of initial CT scans acquired in the first COVID-19 patients presenting in the early phase of the pandemic at our Level-I center between 23 March and 3 April 2020. All patients included were positive for SARS-CoV-2 (RT-PCR of nasopharyngeal and oropharyngeal swab samples); patients with a negative RT-PCR test result were excluded. We recorded the patients’ initial symptoms and clinical course, comorbidities, indications for CT acquisition and treatment with intubation. Involvement of the lungs on CT scans was determined subjectively and semi-quantitative in terms of the extent of consolidations and ground-glass opacities. In addition, secondary findings such as pleural effusion, lymph nodes, or distribution and accentuation of infiltrates were registered. Subjective assessment of the severity of lung involvement was carried out by consensus and estimated as follows: none, minor = 0–33%, moderate = 34–66%, high = 67–100% of total lung area affected by ground-glass opacities and consolidations. For simple semi-quantitative determination of lung involvement, the affected lung area was measured in polygonal regions of interest (ROIs) in one image at three levels (aortic arch, tracheal bifurcation, inferior end of the xiphoid). For each level, the percentage of involved area was calculated by adding up affected areas and dividing the sum by the total lung area in the same image. In addition, the average involved lung area was calculated as the mean of the three measurement levels. Measurement is illustrated in Figure 1.

CT scans were acquired with Canon Aquilion Prime (Tokyo, Japan; voltage 100 kVp, tube current 10–120 mA, Recon IR level AIDR 3D standard, recon section interval 1 mm) and GE Light-Speed VCT (Boston, MA, USA; voltage 100 kVp, tube current 20–120 mA, Recon IR level ASIR 50, recon section interval 0.625 mm). CT scans were then acquired during a single breath-hold and without contrast agent. A low-dose protocol (effective dose < 1 mSv) following our department’s standard for COVID-19 patients was used [10]. Identical parameters were used for all scans. ROIs were defined at 5 mm slice thickness using a lung kernel.

We report absolute and relative frequencies for categorical variables and medians along with interquartile ranges for continuous variables. Results are reported for the total study population as well as for the two clinical endpoints—ICU admission and intubation. Risk factors for the instantaneous risk of intubation and ICU admission were investigated by competing risks Cox regression, treating discharge and death as competing risks. Time was operationalized starting from the date of the CT examination. Characteristics included in these models were sex, age, chronic lung disease, smoking, and percentage of involved lung area on initial CT scans. Results are displayed as cause-specific hazard ratio (HR) estimates along with 95% confidence intervals (CIs). Additionally, diagnostic performance was assessed by receiver operating characteristic (ROC) curves and the area under the ROC curves (AUC). Statistical analysis was performed using R (R Core Team) including additional packages for data handling, plotting, and analysis [11,12,13,14].

## 3. Results

### 3.1. Patient Population

The study population consisted of 28 patients (see Table 1 for a summary of demographic patient data). Most of the patients were brought to the hospital by a rescue service (42.9%, n = 12; median age, 67 years), three patients were referred by general practitioners (10.7%; median age, 71 years), and seven presented themselves to the emergency department (25%; median age, 33 years). As our hospital provides the highest level of care (Level-I center), some patients were transferred from peripheral hospitals due to severe symptoms (21.4%, n = 6; median age, 57 years). The most common indications for CT in our patient population were clinical symptoms such as severe dyspnea (53.6%), followed by assessment of pulmonary involvement of COVID-19 infection (39.3%) and abnormal X-ray findings (3.6%). The median hospital stay was 15 days (range 1–98 days, interquartile range (IQR): 16 days). One patient did not require hospitalization and was therefore discharged into home isolation. Six patients died in hospital (median time to death since positive SARS-CoV-2 PCR test was 15.5 days (range 4–79 days, IQR: 26 days); all other patients were discharged after treatment. The median time between the onset of symptoms and a positive SARS-CoV-2 PCR test was 6 days (range: 0–14 days, IQR: 3.5–10.2 days). CT examinations were performed a median of one day after the positive PCR test result (range: 4 days before to 9 days after PCR test; IQR: 0–4.5 days). The transfer to the intensive care unit was based on clinical parameters (e.g., oxygen saturation, respiratory rate, blood pressure); the result of the CT examination was primarily used to evaluate the course of the disease and was not an indicator for the ICU admission.

The patients had a median age of 61 years (range: 21.9 to 86.0 years; IQR: 49.1–72.0 years), 21.4% were smokers. Patients in the smoking group were younger than in the nonsmoking group (smokers: 55.6 years; nonsmokers: 67.9 years). The most common symptoms at initial presentation were fever, cough, and dyspnea. The 18 patients (64.3%) who had to be transferred to ICU were more often female and younger. Hypertension and obesity were more common in the group of ICU patients than in patients receiving normal care. Fever, limb pain, dyspnea, and abdominal symptoms were more common in ICU patients and intubated patients. 12 patients (42.9%) had to be intubated. Intubated patients had a lower median age than patients without intubation. There was hardly any difference between patients without and with intubation in terms of pre-existing lung conditions (Table 1).

### 3.2. Subjective Estimation of Pulmonary Involvement

In the majority of cases, the severity of pulmonary involvement on CT scans was subjectively classified as moderate. Involvement in those patients later on in need of ICU treatment and intubation was more often classified as moderate or major. The results of subjective chest CT evaluation including imaging features of COVID-19 are summarized in Table 2.

The predominant CT findings were ground-glass opacities and consolidations. Pleural effusions and lymphadenopathy were rare. The distribution pattern of pulmonary infiltrates was mostly peripheral (85.7%, n = 24). Only a few cases showed predominantly central infiltrates (14.3%, n = 4). Basal infiltrates were most common (53.6%), followed by posterolateral (25.0%) and apical infiltrates (14.3%) (see Table 2).

### 3.3. Semi-Quantitative Measurement of Pulmonary Involvement

With the method presented here, we measured a median affected lung area of 20.5%. The largest infiltrates were measured in the basal parts of the lungs (at the level of the xiphoid, median 26.6%). Within the different measurement levels, patients on ICU as well as intubated patients were more severely affected (see Table 2).

**Figure 2:**
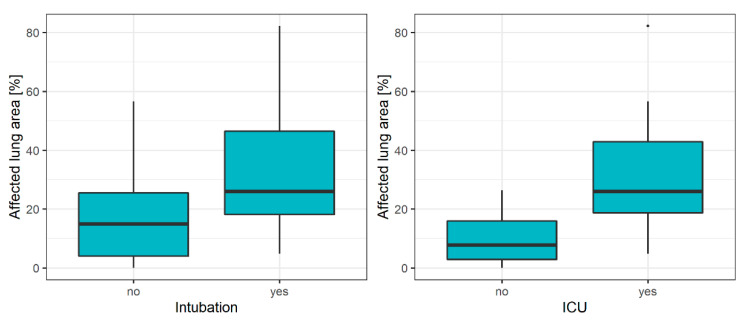
Boxplots of the extent of affected lung area on initial CT in relation to the two clinical endpoints—need of ICU treatment and intubation. Median lung involvement in initial CT scans is more severe in ICU (left) patients compared to patients in conventional wards. The same tendency can be seen for intubated patients (right).

The median affected lung parenchyma area was larger in patients treated in the ICU and in intubated patients (see Figure 2).

**Figure 3:**
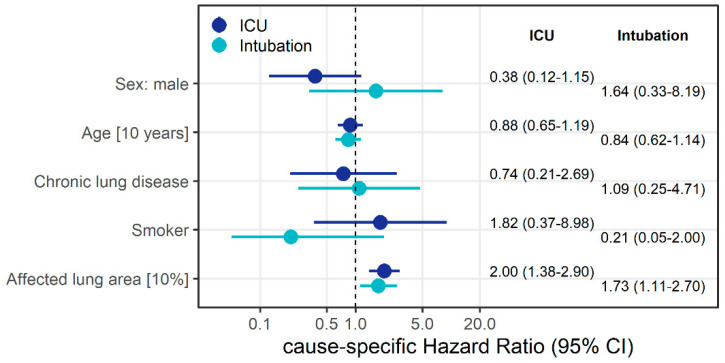
Cause-specific hazard ratio estimates along with 95% confidence intervals (CI) derived from competing risks Cox regression models to identify factors associated with the instantaneous risk of ICU admission and intubation, respectively. Event of interest was intubation; discharge and death were treated as competing risks. All displayed variables were included in the model.

Based on the competing risk model, we found that a 10% increase in affected lung area increased the cause-specific instantaneous risk of intubation independent of other potential risk factors almost two-fold (HR = 1.73; 95% CI: 1.12–2.70), and doubled the instantaneous risk of ICU admission (HR = 2.00 (1.38–2.90) (see Figure 3).

**Figure 4:**
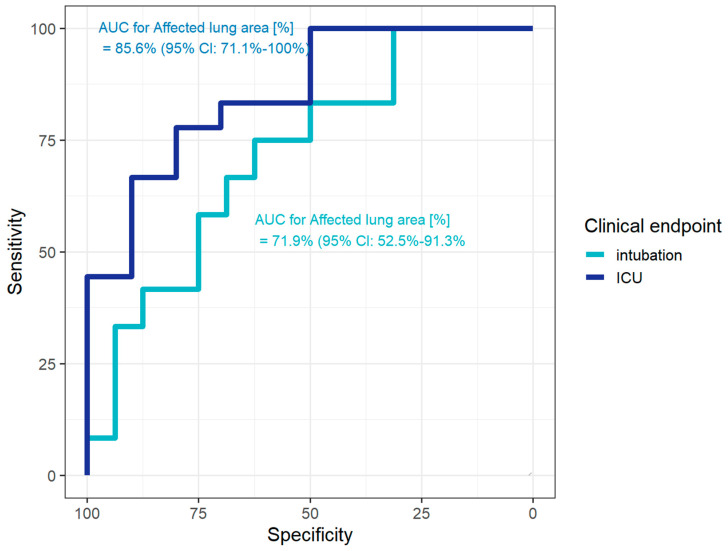
Receiver operating characteristic (ROC) curves to evaluate the diagnostic performance of affected lung area (%) for prediction of intensive care unit (ICU) treatment or intubation in patients with COVID-19. For the endpoint of ICU treatment (dark blue), the area under the curve (AUC) is 85.6% (95% CI: 72.1–100%). For the endpoint of intubation (light blue), the AUC is 71.9% (95% CI: 52.5–91.3%).

In ROC analysis, the AUC for the endpoint of ICU admission was 85.6% (95% CI: 72.1–100%) for the affected lung area. Using Youden’s index, the cutoff of 17.6% of the affected lung parenchyma could be used to differentiate between patients regarding their need of ICU treatment, resulting in a specificity of 80.0% and a sensitivity of 77.8%. For the endpoint of intubation, the AUC was 71.9% (95% CI: 52.5–91.3%) for the affected lung area. Again, based on Youden’s index, the cutoff of 19% of the affected lung area was determined (associated with 62.5% specificity, 75.0% sensitivity) (Figure 4).

**Figure 5:**
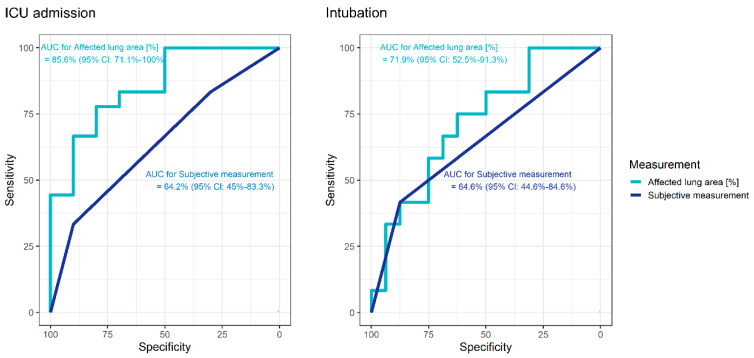
Receiver operating characteristic (ROC) curves to evaluate the diagnostic performance of affected lung area (light blue) and the subjective assessment (dark blue) for prediction of intensive care unit (ICU) treatment in patients with COVID-19 (left). Receiver operating characteristic (ROC) curves to evaluate the diagnostic performance of affected lung area (light blue) and the subjective assessment (dark blue) for prediction of intubation in patients with COVID-19 (right).

In ROC analysis, the AUC for the endpoint of ICU admission was 64.2% (95% CI: 45–83.3%) and for intubation 64.6% (95% CI: 44.6–84.6%) for the subjective assessment (see Figure 5).

**Figure A1:**
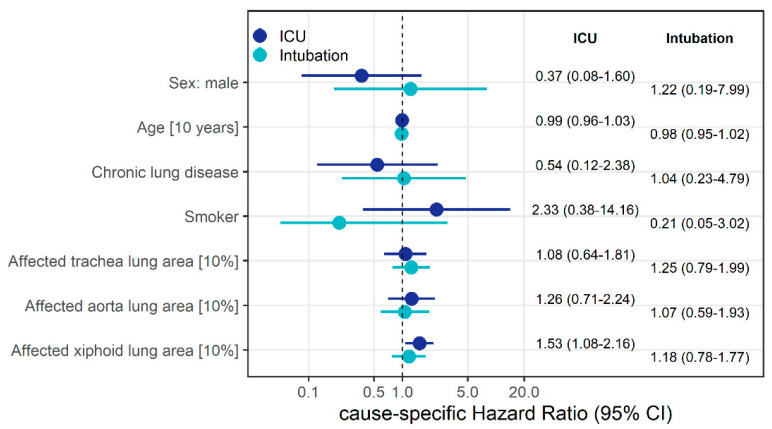
Cause-specific hazard ratio estimates along with 95% confidence intervals (CI) derived from competing risks Cox regression models to identify factors influencing the instantaneous risk of intubation. Events of interest were ICU admission and intubation, respectively; discharge was treated as a competing risk. All displayed variables were included in the model.

**Figure A2:**
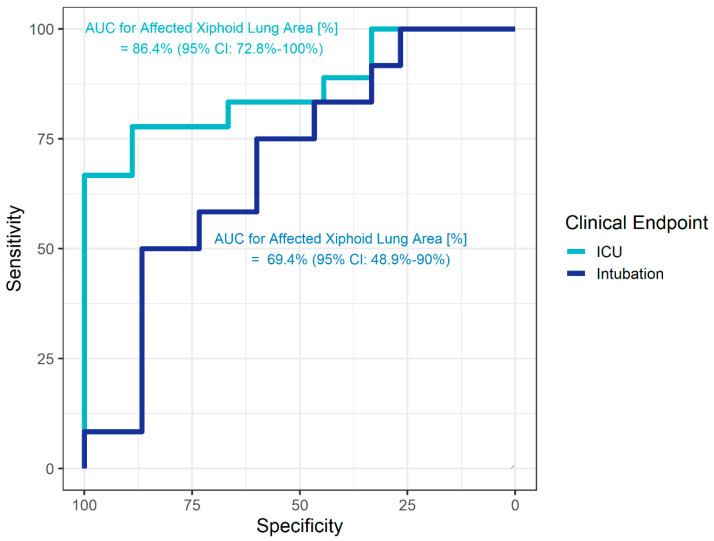
Diagnostic performance for prediction of intensive care unit (ICU, dark blue) treatment or intubation (light blue) in patients with COVID-19. Receiver-operating characteristic (ROC) curves based on the affected lung area on the level of the xiphoid (%).

Solely based on the measurement of the affected lung area on the level of the xiphoid, we found that a 10% increase in affected xiphoid lung area was associated with approximately a 1.2-fold increased instantaneous risk of intubation, independent of other potential risk factors (HR = 1.18; 95% CI: 0.78–1.77), and also associated with a higher risk of ICU admission (HR = 1.53 CI: 1.08–2.16) (see Figure A1 in Appendix A). Based on the sole measurement of the affected lung area on the level of the xiphoid, the AUC for the endpoint ICU admission was 86.4% (95% CI: 72.8–100%) and for the endpoint intubation 69.4% (95% CI: 48.9–100%) (see also Figure A2 in Appendix A).

## 4. Discussion

The aim of our study was to investigate whether a simple semi-quantitative measurement of the extent of pulmonary involvement on initial chest CT scans of patients with COVID-19 can help in identifying patients in subsequent need of ICU treatment and intubation. Earlier studies have shown that imaging-based quantitative assessment of the severity of lung involvement is possible using deep learning or volumetry [5,8]. Deep learning is not yet an established procedure, and many deep-learning models still require involvement of a radiologist [5]. For accurate volumetry, additional clinical parameters, such as Body-Mass-Index (BMI) or spirometry results, are needed [8]. However, these methods are not widely available, especially in view of rising prevalence rates in low- and middle-income countries, a simple method is needed.

The pulmonary CT findings of COVID-19 disease described in the literature include bilateral, peripherally accentuated consolidations and ground-glass opacities (GGOs), and are confirmed by our results [7,15,16]. As described in the literature, GGOs and consolidations are more common in ICU patients and intubated patients [5]. Pleural effusions and lymphadenopathy occurred in our patients but are rare findings [7,15,16]. In our patients, the changes caused by COVID-19 most commonly affected the basal parts of the lungs. This supports published observations that, in addition to a peripheral distribution pattern, the basal pulmonary segments are most markedly affected [17].

The clinical presentation of COVID-19 infection is heterogeneous with up to 17% of patients requiring ventilation [5]. The most common symptoms of patients infected with SARS-CoV-2 in our study were cough and fever, which is consistent with reports in the literature [18]. Additionally, in line with previous research is the high proportion of men in our study and that 7.1% of patients needed extracorporeal membrane oxygenation (ECMO), which is within the published range (6–43%) [4]. 64.3% of patients included in our analysis were treated in the ICU; in one of the first studies from China, 31% were treated at the ICU [18]. This discrepancy is probably due to the fact that our hospital is a Level-I center, which means that we also admit patients with severe disease from other hospitals.

Chronic lung disease, which is assumed to be a potential risk factor for severe COVID-19, was not significantly related to subsequent ICU treatment or intubation in our analysis. This is probably due to the small sample size and patient selection (see above) [4,19,20]. However, results from the literature support our observation that overweight patients need to be transferred to the ICU more often [21].

In our study, patients who had to be transferred to the ICU and be intubated had more extensive pulmonary involvement based on semi-quantitative measurement, which is consistent with earlier findings [8]. Colombi et al. for example showed that both visually estimated and objectively quantified pulmonary involvement using a software-based technique were helpful in identifying patients in need of ICU treatment [8]. The authors reported that the odds of being transferred to the ICU increased with an odds ratio of 5.4 when less than 73% of the lung parenchyma was well aerated, which is consistent with our results. Our data indicate that the extent of the affected lung parenchyma area in CT is associated with the subsequent need of ICU treatment and intubation, independent of age, sex, smoking, or pre-existing lung disease. More specifically, a 10% increase in affected lung area almost doubled the cause-specific instantaneous risk of intubation, independent of other potential risk factors (HR = 1.73; 95% CI: 1.12–2.70), and similarly increased the instantaneous risk of ICU admission (HR = 2.00; 1.38–2.90).

Although having evaluated the diagnostic ability of the lung parenchyma in our study, we deem our sample as too small and potentially not representative to define a reliable cutoff.

In our analysis, the semi-quantitative measurement outperformed the subjective assessment with regard to the diagnostic ability. Colombi et al. also conclude that subjective assessment is inferior to semi-quantitative assessment [8]. Interestingly, the sole measurement at the level of the xiphoid had a comparable diagnostic ability for ICU admission and intubation as all three measurements, making the measurement simpler and faster.

Our study is limited by a small sample size, a short inclusion period and the exclusive use of initial CT scans. Due to its observational nature it includes a selection of patients who might not be representative of the general population of COVID-19 patients requiring treatment at a hospital.

Due to the very dynamic situation, we limited our sample to the first patients who were tested positive for COVID-19 and were examined by chest CT in our hospital until 3 April 2020. While the number of patients is small, we could follow their entire clinical course: six patients died in hospital, all others were discharged. Patient characteristics during that early phase of the COVID-19 pandemic may be different from those of patients presenting during the current, second wave. The sample size is small; therefore, our results need to be confirmed in a larger number of patients. Published studies suggest that lung involvement changes in the course of the disease [5]. However, since many hospitals now perform a CT scan upon admission of patients with known or suspected COVID-19, this initial scan has the greatest importance for the prediction of the clinical course [16,22]. Placement of ROIs was performed manually and is therefore somewhat subjective. On the other hand, we used standardized levels of measurement and a predefined slice thickness of CT scans to ensure comparability between patients. Detection of abnormal lung tissue, e.g., by means of density thresholds, would probably be more objective, and the use of such methods has already been described in the literature [10]. However, the primary aim of our study was to investigate whether comparable and reliable results could be obtained using a simple and widely available method. As already mentioned, we saw a high proportion of patients with severe COVID-19, which is not representative of the general population of patients with the disease. We observed that especially young, smoking patients with severe courses were transferred to our center from other hospitals. This patient selection led to the seeming paradoxical results based on our small sample that higher age and smoking tended to have a protective effect for intubation and transfer to the ICU, which contradicts the general assumption that higher age and smoking are risk factors for a severe course of COVID-19 [20,23].

## 5. Conclusions

In conclusion, our results confirm the typical pulmonary CT findings of COVID-19 described in the literature. The simple semi-quantitative measurement of the affected lung parenchyma area at three levels as presented here could contribute to a more reliable prediction of the need of ICU treatment and intubation. This could help to optimize the use of available intensive care/ventilation resources in light of the global COVID-19 pandemic.

## Figures and Tables

**Table 1:** Summary of demographic and other patient data by endpoints and in the total study population.

	Patients without ICU Admission (n = 10)	Patients Requiring ICU Admission (n = 18)	Patients without Intubation (n = 16)	Patients with Intubation (n = 12)	Total (n = 28)
**Sex**					
Female	2 (20.0%)	7 (38.9%)	6 (37.5%)	3 (25.0%)	9 (32.1%)
Male	8 (80.0%)	11 (61.1%)	10 (62.5%)	9 (75.0%)	19 (67.9%)
Age (median, IQR)	65.9 (54.0–71.5)	58.2 (39.6–74.9)	65.9 (53.9–72.4)	56.2 (34.8–68.0)	61.0 (49.1–72.0)
**Comorbidities**					
Diabetes	3 (30.0%)	3 (16.7%)	3 (18.8%)	3 (25.0%)	6 (21.4%)
Smoking	3 (30.0%)	3 (16.7%)	5 (31.2%)	1 (8.3%)	6 (21.4%)
Alcohol abuse	2 (20.0%)	0 (0.0%)	2 (12.5%)	0 (0.0%)	2 (7.1%)
Hypertension	5 (50.0%)	9 (50.0%)	6 (37.5%)	8 (66.7%)	14 (50.0%)
CAD	1 (10.0%)	0 (0.0%)	1 (6.2%)	0 (0.0%)	1 (3.6%)
Chronic heart failure	1 (10.0%)	1 (5.6%)	1 (6.2%)	1 (8.3%)	2 (7.1%)
Obesity	1 (10.0%)	4 (22.2%)	1 (6.2%)	4 (33.3%)	5 (17.9%)
Chronic lung disease	5 (50.0%)	10 (55.6%)	9 (56.2%)	6 (50.0%)	15 (53.6%)
Bronchiectasis	1 (10.0%)	3 (16.7%)	2 (12.5%)	2 (16.7%)	4 (14.3%)
Emphysema	4 (40.0%)	5 (27.8%)	6 (37.5%)	3 (25.0%)	9 (32.1%)
Fibrosis	0 (0.0%)	2 (11.1%)	1 (6.2%)	1 (8.3%)	2 (7.1%)
**Symptoms**					
Weakness	7 (70.0%)	10 (55.6%)	9 (56.2%)	8 (66.7%)	17 (60.7%)
Limb pain	3 (30.0%)	8 (44.4%)	5 (31.2%)	6 (50.0%)	11 (39.3%)
Fever	5 (50.0%)	15 (83.3%)	9 (56.2%)	11 (91.7%)	20 (71.4%)
Cough	8 (80.0%)	12 (66.7%)	11 (68.8%)	9 (75.0%)	20 (71.4%)
Dyspnea	7 (70.0%)	14 (77.8%)	10 (62.5%)	11 (91.7%)	21 (75.0%)
Abdominal symptoms ^1^	1 (10.0%)	5 (27.8%)	2 (12.5%)	4 (33.3%)	6 (21.4%)
Cardiac symptoms	3 (30.0%)	2 (11.1%)	4 (25.0%)	1 (8.3%)	5 (17.9%)
**Type of ventilation**					
No	4 (40.0%)	0 (0.0%)	4 (25.0%)	0 (0.0%)	4 (14.3%)
Oxygen	6 (60.0%)	0 (0.0%)	6 (37.5%)	0 (0.0%)	6 (21.4%)
Noninvasive ^2^	0 (0.0%)	6 (33.3%)	6 (37.5%)	0 (0.0%)	6 (21.4%)
Invasive ^3^	0 (0.0%)	8 (44.4%)	0 (0.0%)	8 (66.7%)	8 (28.6%)
ECMO	0 (0.0%)	4 (22.2%)		4 (33.3%)	4 (14.3%)

^1^ Abdominal symptoms: abdominal pain, diarrhea, nausea, cardiac symptoms: chest pain, angina pectoris. Oxygen—administration of oxygen through nasal cannula. ^2^ Noninvasive—noninvasive ventilation via CPAP or high flow. ^3^ Invasive—intubation performed for invasive ventilation. Abbreviations: ICU—intensive care unit. ECMO—extracorporeal membrane oxygenation. CAD—coronary artery disease. IQR—interquartile range. CPAP- continuous positive airway pressure.

**Table 2:** Results of subjective and objective CT evaluation.

	Patients without ICU Admission (n = 10)	Patients Requiring ICU Admission (n = 18)	Patients without Intubation (n = 16)	Patients with Intubation (n = 12)	Total (n = 28)
**Subjective estimate of extent of pulmonary involvement**					
None	0 (0.0%)	0 (0.0%)	0 (0.0%)	0 (0.0%)	0 (0.0%)
Minor	3 (30.0%)	3 (16.7%)	4 (25.0%)	2 (16.7%)	6 (21.4%)
Moderate	6 (60.0%)	9 (50.0%)	10 (62.5%)	5 (41.7%)	15 (53.6%)
Major	1 (10.0%)	6 (33.3%)	2 (12.5%)	5 (41.7%)	7 (25.0%)
**Semi-quantitative measurement of pulmonary involvement**					
Aortic arch (%)	5.4 (2.7–14.5)	13.2 (2.2–42.9)	6.7 (1.9–15.6)	22.3 (3.4–43.5)	11.3 (2.1–32.9)
Tracheal bifurcation (%)	2.9 (2.1–10.8)	18.2 (10.6–48.6)	8.0 (2.6–18.3)	18.7 (8.9–49.6	12.0 (2.7–34.8)
Inferior end of the xiphoid (%)	12.8 (2.7–21.4)	35.1 (26.5–51.0)	21.4 (7.2–33.7)	34.5 (25.1–46.2)	26.6 (10.9–37.5)
Affected lung area (%)	7.8 (2.8–15.9)	26.0 (18.7–42.9)	15.0 (4.0–25.5)	26.0 (18.2–46.5)	20.4 (6.1–32.6)
**CT findings**					
Consolidations					
None	3 (30%)	1(5.6%)	3 (18.8%)	1 (8.3%)	4 (14.3%)
Minor ^1^	4 (40.0%)	1 (5.6%)	7 (43.8%)	4 (33.3%)	11 (39.3%)
Moderate ^1^	2 (20.0%)	7 (38.9%)	5 (31.3%)	4 (44.4%)	9 (32.1%)
Major ^1^	1 (10.0%)	3 (16.7%)	1 (6.3%)	3 (25.0%)	4 (14.3%)
Ground-glass opacities					
None	0 (0.0%)	2 (11.1%)	1 (6.3%)	1 (8.3%)	2 (7.1%)
Minor ^1^	7 (53.8%)	6 (46.2%)	8 (50.0%)	5 (38.5%)	13 (100.0%)
Moderate	3 (30.0%)	9 (50.0%)	7 (43.8%)	5 (41.7%)	12 (42.9%)
Major	0 (0.0%)	1 (5.6%)	0 (0.0%)	1 (8.3%)	1 (3.6%)
Pleural effusions					
None	9 (90.0%)	15 (83.3%)	14 (87.5%)	10 (83.3%)	24 (85.7%)
Minor ^1^	0 (0.0%)	2 (11.1%)	1 (6.3%)	1 (8.3%)	2 (7.1%)
Moderate ^1^	1 (10.0%)	0 (0.0%)	1 (6.3%)	0 (0.0%)	1 (3.6%)
Major ^1^	0 (0.0%)	1 (5.6%)	0 (0.0%)	1 (8.3%)	1 (3.6%)
Lymphadenopathy	2 (20.0%)	2 (11.1%)	3 (18.8%)	1 (8.3%)	4 (14.3%)

^1^ Classification of severity of findings: minor = 0–33%, moderate = 34–66, major = 67–100%. Affected lung area was calculated from all three measured values.
